# A statistical simulation model for field testing of non-target organisms in environmental risk assessment of genetically modified plants

**DOI:** 10.1002/ece3.1019

**Published:** 2014-03-15

**Authors:** Paul W Goedhart, Hilko van der Voet, Ferdinando Baldacchino, Salvatore Arpaia

**Affiliations:** 1 Biometris, Plant Research International, Wageningen University and Research CentreP.O. Box 16, 6700 AA, Wageningen, The Netherlands; 2 ENEA: National Agency for New Technologies, Energy and Sustainable Economic Development, Centro Ricerche TrisaiaS.S. 106 Ionica, 75026, Rotondella, Italy

**Keywords:** Difference testing, environmental risk assessment, equivalence testing, field trials, simulation model, statistical distributions, statistical power

## Abstract

Genetic modification of plants may result in unintended effects causing potentially adverse effects on the environment. A comparative safety assessment is therefore required by authorities, such as the European Food Safety Authority, in which the genetically modified plant is compared with its conventional counterpart. Part of the environmental risk assessment is a comparative field experiment in which the effect on non-target organisms is compared. Statistical analysis of such trials come in two flavors: difference testing and equivalence testing. It is important to know the statistical properties of these, for example, the power to detect environmental change of a given magnitude, before the start of an experiment. Such prospective power analysis can best be studied by means of a statistical simulation model. This paper describes a general framework for simulating data typically encountered in environmental risk assessment of genetically modified plants. The simulation model, available as Supplementary Material, can be used to generate count data having different statistical distributions possibly with excess-zeros. In addition the model employs completely randomized or randomized block experiments, can be used to simulate single or multiple trials across environments, enables genotype by environment interaction by adding random variety effects, and finally includes repeated measures in time following a constant, linear or quadratic pattern in time possibly with some form of autocorrelation. The model also allows to add a set of reference varieties to the GM plants and its comparator to assess the natural variation which can then be used to set limits of concern for equivalence testing. The different count distributions are described in some detail and some examples of how to use the simulation model to study various aspects, including a prospective power analysis, are provided.

## Introduction

An essential element in the environmental risk assessment (ERA) of genetically modified (GM) plants is a comparative field trial in which the effect on non–target organisms (NTO), such as aphids, beetles and bumble bees is compared. Such an experiment ensures that the GM plant and its comparator(s) are grown under the same management and environmental conditions, thus enabling a fair and objective comparison. A basic statistical approach for designing and analyzing such field experiments has been outlined in an EFSA guidance document (EFSA, 2010), in Perry et al. (2009) and Semenov et al. (2013). However, in practice the power of these experiments to detect environmental changes of a given magnitude is often unknown, partly because insufficient prior thought is given to what exact endpoints the experiments are supposed to test, and partly because the complex nature of ecological data complicates the power calculations. One of the aims in the EU-funded project “Assessing and Monitoring the Impacts of Genetically modified plants on Agro-ecosystems” (AMIGA) is to devise statistically well-based protocols for the design and analysis of field trials. To prepare this action an inventory was made of existing field studies in the literature, and a statistical simulation model was developed to mimic ecological data such as found in practice. The aim of the current paper is to describe this statistical simulation model and to show how this can be used in the design of field experiments.

Data collection in experimental fields with genetically modified crops has been conducted for many years and a large variability of experimental designs, sampling techniques, guilds of non-target arthropods and statistical methods have been used (e.g., Marvier et al., 2007). To summarize the different approaches presented in the scientific literature, a non-exhaustive inventory of 33 field studies with insect resistance transgenic plants was compiled among those firstly published, where the detection of possible effects of GM plants on natural enemies was the primary goal of the study (Table 1). The papers were published from 1992 until 2005. This time-span was chosen to include the very first published experiments of this kind, and also to incorporate the first available data from surveys in GM commercial fields. Different crops were included in the selection. The table presents some of the indicators relevant to the experimental design, collection methods and statistical analyses performed on the data. None of the papers provided a prospective power analysis for the experiments described.

**Table 1:** Main characteristics of fields experiment 1992–2005 using GM crops.

Authors and Journal	Functional group	Crop	Measurement endpoint	Dimensions	Experimental design	Statistical method
Johnson & Gould (1992)Environ. Entomol.	Parasitoids	Tobacco	Parasitism rate	9 replications, 2 years	Randomized blocks	Chi-square
Johnson (1997)Environ. Entomol.	Parasitoids	Tobacco	Parasitism rate	3 years, 15 sites	Randomized blocks	ANOVA
Mascarenhas and Luttrell (1997) Environ. Entomol.	Parasitoids	Cotton	Host survival	4 replications	Completely randomized	ANOVA
Orr & Landis (1997) J. Econom. Entomol.	Parasitoids,Predators	Maize	Egg fate, Parasitism rate, Visual counts	3 replications (50 plants), 3 sampling dates	Completely randomized	ANOVA
Pilcher et al. (1997)Environ. Entomol.	Predators	Maize	Abundance, Visual counts	2 years, 3 replications (6 plants in each), 3 sampling dates	Randomized blocks	ANOVA
Riddick et al. (1998) Ann. Entomol. Soc. Am.	Predators	Potato	Abundance, Visual counts, Sweep nets, Pitfall traps	2 years, 3 sites	Completely randomized	ANOVA
Buckelew et al. (2000) J. Econom. Entomol.	Predators	Soybean	Abundance, Sweep nets	2 sites, 2 years, weekly samplings	Randomized blocks	ANOVA
Al–Deeb et al. (2001) J. Econom. Entomol.	Predators	Maize	Abundance, Visual counts	40 plants, 2 locations	Completely randomized	ANOVA mixed model
Reed et al. (2001)Entomol. Exp. Appl.	Predators	Potato	Abundance, Visual counts	2 years, 6 replications	Latin square	ANOVA
Wold et al. (2001) J. Entomol. Science	Predators	Maize	Abundance, Visual counts	2 years, 4 replications, 6 sampling dates	Completely randomized	ANOVA
Bourguet et al. (2002)Environ. Biosaf. Res.	Predators, Parasitoids	Maize	Abundance,Parasitization	2 sites, 4 replications, weekly samplings	Split-plot	ANOVA
Manachini & Lozzia (2002) Boll. Zool. Agr. Bachic.	Soil organisms	Maize	Abundance, Diversity	2 separate fields, 8 locations50 soil samples	n.a.	ANOVA
Al–Deeb and Wilde (2003) Environ. Entomol.	Predators	Maize	Abundance, Visual counts, Pitfall traps	2 years, 8 locations	Completely randomized	ANOVA mixed model
Jasinski et al. (2003)Environ. Entomol.	Predators	Soybean,Maize	Abundance, Sweep nets, Sticky traps, Soil samples	24 commercial fields	n.a.	ANOVA
Men et al. (2003)Environ. Entomol.	Herbivores,Predators,Parasitoids	Cotton	Abundance, Sweep nets, Visual counts	3 years, 3 replications, 5 sampling dates	Completely randomized	ANOVA, Diversity indices
Musser & Shelton (2003) J. Econom. Entomol.	Predators	Maize	Abundance, Egg predation	2 years 2–10 plants/replication	Randomized block	ANOVA
Volkmar et al. (2003)Agric. Ecosys. Environ.	Predators	Sugar beet	Abundance, Pitfall Traps	4 replications	Randomised block	ANOVA
Wu & Guo (2003)Environ. Entomol.	Predators	Cotton	Abundance, Visual counts	3 replications	Completely randomized	ANOVA
Candolfi et al. (2004)Biocontrol. Sci. Techn.	Predators,HerbivoresSoil org.,	Maize	Abundance, Pitfall traps, Yellow traps	3 replications, field size	Completely randomized	Principal response curves, Diversity indices
Duan et al. (2004)Environ. Entomol.	Predators	Potato	Abundance, Pitfall traps	2 years, 6 replications	Latin square	ANOVA
Manachini et al. (2004) IOBC/WPRS Bullettin	Soil organisms	Canola	Extraction from soil	3 replications	Completely randomized	Multivariate
Wade French et al. (2004) Environ. Entomol.	Predators	Maize	Abundance, Pitfall traps	2 years, commercial fields	n.a.	Canonical correspondence
Wei-Di et al. (2004)Chinese J. Agric. Biotec.	Herbivores, Predators,Parasitoids	Cotton	Abundance, Diversity	2 years, 3 replications	Completely randomized	ANOVA, Diversity indices
Bhatti et al. (2005a)Environ. Entomol.	Predators,Detritivores,Soil herbivore	Maize	Abundance	3 years	Split-plot	ANOVA mixed model
Bhatti et al. (2005b)Environ. Entomol.	Predators,Herbivores,Parasitoids	Maize	Abundance	3 years two-weekly samplings	Split-plot	ANOVA mixed model
Daly & Buntin (2005)Environ. Entomol.	Predators,Herbivores	Maize	Abundance	2 locations 2 years, 4 replications, weekly samplings	Completely randomized	ANOVA mixed model
De La Poza et al. (2005)Crop Protection	Predators	Maize	Abundance, Visual counts, Pitfall traps	2 locations, 3 years, 3–4 replicates	Completely randomized(split for year and location)	ANOVA
Hagerty et al. (2005)Environ. Entomol.	Predators,Herbivores	Cotton	Abundance, Damage	2 years, 4 replications	Completely randomized	ANOVA
Head et al. (2005)Environ. Entomol.	Predators,Herbivores	Cotton	Abundance,Predation rates	3 years, 3–4 replications,6–16 sampling dates	Completely randomized	ANOVA mixed model with repeated measures
Naranjo (2005)Environ. Entomol.	Predators	Cotton	Diversity	6 years, 3–4 replications	Completely randomized	ANOVA, PCA
Pons et al. (2005)European J. Entomol.	Herbivores	Maize	Pest incidence	3 years, 4 replications, various sampling dates	Completely randomized(year as factorial element)	ANOVA
Torres and Ruberson (2005) Environ. Entomol.	Predators	Cotton	Abundance	3 years, 3 replicates, weekly samplings	Completely randomized	ANOVA mixed model with repeated measures
Whitehouse et al. (2005) Environ. Entomol.	Different guilds	Cotton	Diversity Index	3 years, 2–3 replicates, weekly samplings	Completely randomized	ANOVA mixed model with repeated measures

Field trials are thus diverse, but an example shows some typical elements. Al–Deeb and Wilde (2003) describe experiments to test the effects of the Cry3Bb1 toxin in Bt corn on aboveground non–target arthropods. The experiments were performed on eight locations in one year and three locations in a second year, and they involved three GM varieties and two isolines in combination with up to nine different seed and spraying treatments. Randomized complete block designs were used with 2–4 blocks and 8–40 plots. Visual inspection provided count data on 15–20 plants per plot. Average counts per plant for five NTOs varied between 0 and 70. Pitfall trap count data observed at 3–7 time points were reported as average numbers per pitfall trap between 0 and 616 for eight NTOs. Based on a statistical analysis using analysis of variance the authors concluded that no significant differences in numbers were detected between Bt corn and its non–Bt isoline. However, there is no mention of the effect sizes that these experiments would have been able to detect with a reasonable statistical power. In fact, the data provided are insufficient to draw any conclusion on the statistical power of the performed experiments, and this is also the case for many other reported studies. Indeed, in a few cases the importance of such an analysis had been singled out (Andow, 2003) and attempts to design field experiments on such bases were done in rare cases (e.g., Squire et al., 2003; Duan et al., 2006). To improve this situation the EFSA guidance asks for prospective power analyses to be performed. This issue is further developed in the present paper.

Typical data in environmental risk assessment of GM plants are counts or presence/absence data of NTOs. The basic distribution for counts is the Poisson distribution, while presence/absence data can usually be modeled by a binomial distribution. Clumping of individuals might give rise to an overdispersed distribution such as the negative binomial for counts and the beta-binomial distribution for presence/absence data. Also the number of zero observations can be larger than predicted by the distribution and this gives rise to so-called excess-zero distributions. In many experiments, NTOs are sampled at different points in time, for example weekly, for all experimental units. The data are thus repeated measurements probably with some form of autocorrelation across time within experimental units. Depending on the species various patterns across time are possible. Moreover experiments are frequently repeated on different locations and in different years.

The statistical analysis of ERA field trials comes in two flavors: difference testing and equivalence testing (van der Voet et al., 2011). The aim of the difference test is to reject the null hypothesis of no difference between the GM plant and its comparator. A significant difference test is then a “proof of difference”, but this does not state that the difference is biologically relevant and constitutes a true hazard to the environment. Poorly designed experiments with low levels of replication may have low statistical power of finding a true difference. So the absence of a significant difference is not a proof that there is no difference, or “absence of evidence is not evidence of absence” (Altman and Bland, 1995). An equivalence test on the other hand employs a null hypothesis of non-equivalence, that is, that the difference between the GM plant and its comparator is larger than some pre-described equivalence limit, also called limit of concern (LOC). Rejection of the non-equivalence hypothesis implies that the difference is smaller than the LOC and this can be regarded as a “proof of safety”. The advantage of equivalence testing is therefore that the onus is placed back on to those who wish to demonstrate the safety of GMOs to do high quality, well-replicated experiments with sufficient statistical power (Perry et al., 2009). Note that both the difference and equivalence test can be implemented by constructing a single confidence interval for the difference between the GM plant and its comparator. This employs the two one-sided tests (TOST) approach of Schuirmann (1987) for equivalence testing.

It is important to know the statistical properties of difference and equivalence tests, for example the power and robustness of a test and whether the test has the assumed significance level. Such properties are well-known for single experiments using tests based on the normal distribution, such as *t*-tests. For non-normal distributions, small sample properties of difference and equivalence tests are not straightforward. A simulation approach for sample size calculations for a difference test is employed by many authors, for example, Shieh (2001) and Hrdličková (2006) for the Poisson distribution, Shieh (2001) and Demidenko (2008) for the binomial distribution, Aban et al. (2009) and Friede and Schmidli (2010) for the negative binomial distribution. A general practical approach to computing power for non-normal distributions is given by Lyles et al. (2007). However field testing of environmental effects of GM plants on NTOs is much more complicated as it may not only involve non-normal distributions, potentially with excess-zeros, but also a set of reference varieties in addition to the GM plant and its comparator, randomized blocks within an experiment, multiple experiments across different sites and/or years with possibly genotype by environment interaction, and finally repeated measures in time exhibiting some pattern in time possibly with autocorrelation. The object of this paper is to formalize all these elements in a single statistical simulation model which provides a framework for studying various statistical approaches for data analysis of such experiments. The simulation model was implemented in a user-friendly C# program, using the R package (R Core Team, 2012) for simulating from various distributions. The software is available as Supplementary Material to this paper.

This paper first summarizes potentially useful statistical distributions for ecological data. Then the other elements of the statistical simulation model are described, namely block effects, additional varieties, repeated measurements and multiple trials. Some applications of the simulation model for power analysis are described, and possibilities for use and future research needs are discussed.

## Statistical Distributions for Counts

**Figure 1:**
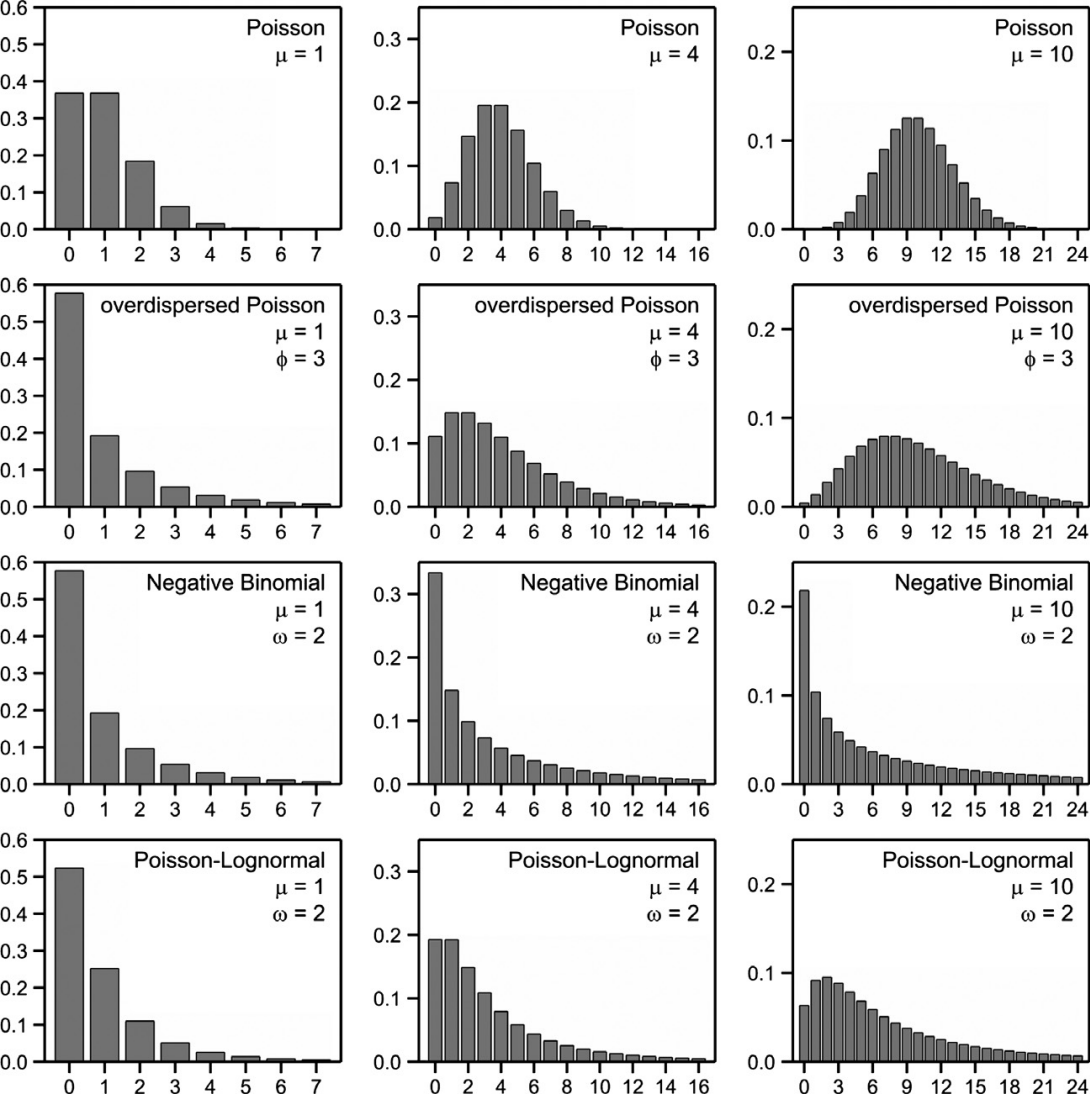
Examples of probabilities of statistical distributions for counts for means *μ* = 1, 4, and 10. The variance of the overdispersed Poisson distribution equals *ϕμ*. The variance of the negative binomial and Poisson-lognormal equals *μ *+ *ωμ*^2^.

The basic distribution for counts is the Poisson distribution. The Poisson distribution arises when events occur independently of each other but at a fixed rate in time or space. The number of events in a fixed time- or space-interval then follows a Poisson distribution. The theoretical variance of the Poisson distribution equals the mean *μ*. Examples of three Poisson distributions are given in Figure 1. The Poisson distribution assumes a fixed rate of events in time or space. However frequently this rate might vary in different time- or space-intervals. A common way to model this is to assume inter-subject variability, also called mixing. It is then assumed that a count *X* follows a Poisson distribution with mean *Z*, where *Z* itself is a random variable with mean *μ* and variance say *τ*^2^. The marginal mean of the distribution of *X* is then given by *μ* and the variance equals *μ *+ *τ*^2^. Consequently the resulting distribution has a variance which is larger than the mean and this is termed over-dispersion. Three common ways to specify the mixing distribution of *Z* result in the overdispersed Poisson distribution, the negative binomial distribution and the Poisson-Lognormal distribution. These are described below.

The overdispersed Poisson distribution arises when *Z* follows a gamma distribution with variance *τ*^2^ = (*ϕ*−1) *μ* which is proportional to the mean *μ*. The resulting distribution is a special form of the negative binomial distribution, see McCullagh and Nelder (1989), with variance *ϕμ* which is also proportional to the mean. Figure 1 shows some examples of the overdispersed Poisson distribution. The so-called quasi likelihood approach is commonly used to fit this distribution. This employs the Poisson likelihood, estimates the dispersion parameter *ϕ* by means of Pearson Chi–squared statistic and adjusts standard errors of estimates accordingly (McCullagh and Nelder, 1989).

The negative binomial distribution arises when the mixing distribution *Z* follows a gamma distribution with mean *μ* and variance *ωμ*^2^. The marginal mean of *X* is then again *μ* and the variance equals *μ *+ *ωμ*^2^. Some examples of the negative binomial distribution are given in Figure 1. This shows that the negative binomial distribution, with a large dispersion parameter *ω*, has a large zero probability and a rather flat tail.

Specification of a lognormal distribution for *Z*, with say mean *λ* and variance *σ*^2^, results in the so-called Poisson-Lognormal distribution. This is equivalent to the introduction of a normally distributed random effect on the scale of the linear predictor in Poisson regression, see Breslow (1984). The mean of the marginal distribution is given by 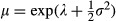 and the variance by *μ* + [exp(*σ*^2^)−1)]*μ*^2^. This is thus the same variance function as the negative binomial distribution with *ω* = [exp(*σ*^2^)−1]. Despite this the distributions can be quite different for large *μ* and *ω* as is shown in Figure 1.

A different approach was introduced by Taylor (1961) who proposed the power relationship *V *= *αμ*^*β*^ between the variance *V* and the mean *μ* for field population counts. This pioneering paper was followed by a series of papers, notably Taylor et al. (1978, 1980), in which it was shown that this relationship fitted well for many species, with varying values of *α* and *β* depending on the species at hand. The relationship was subsequently termed Taylor's power law by some authors. Perry et al. (2003) and Clark et al. (2006) advocate the use of the power law for analyses of count data obtained in farm scale evaluations of GM herbicide-tolerant crops. They found that median values of the power *β*, when considering groups of indicator species, all fall between 1.5 and 2.0, averaging 1.7 overall. There is no statistical distribution associated with Taylor's power law, as it only specifies a relationship between the variance and the mean. Perry et al. (2003) used the negative binomial distribution to simulate according to Taylor's power law by solving *ω* from *μ *+ *ωμ*^2^ = *αμ*^*β*^ for given values of *μ*,*α* and *β*. Using the negative binomial is however arbitrary, as, for example, the Poisson-Lognormal has the same variance to mean relationship, but has a different distribution.

## Statistical Distributions for Presence/Absence Data

**Figure 2:**
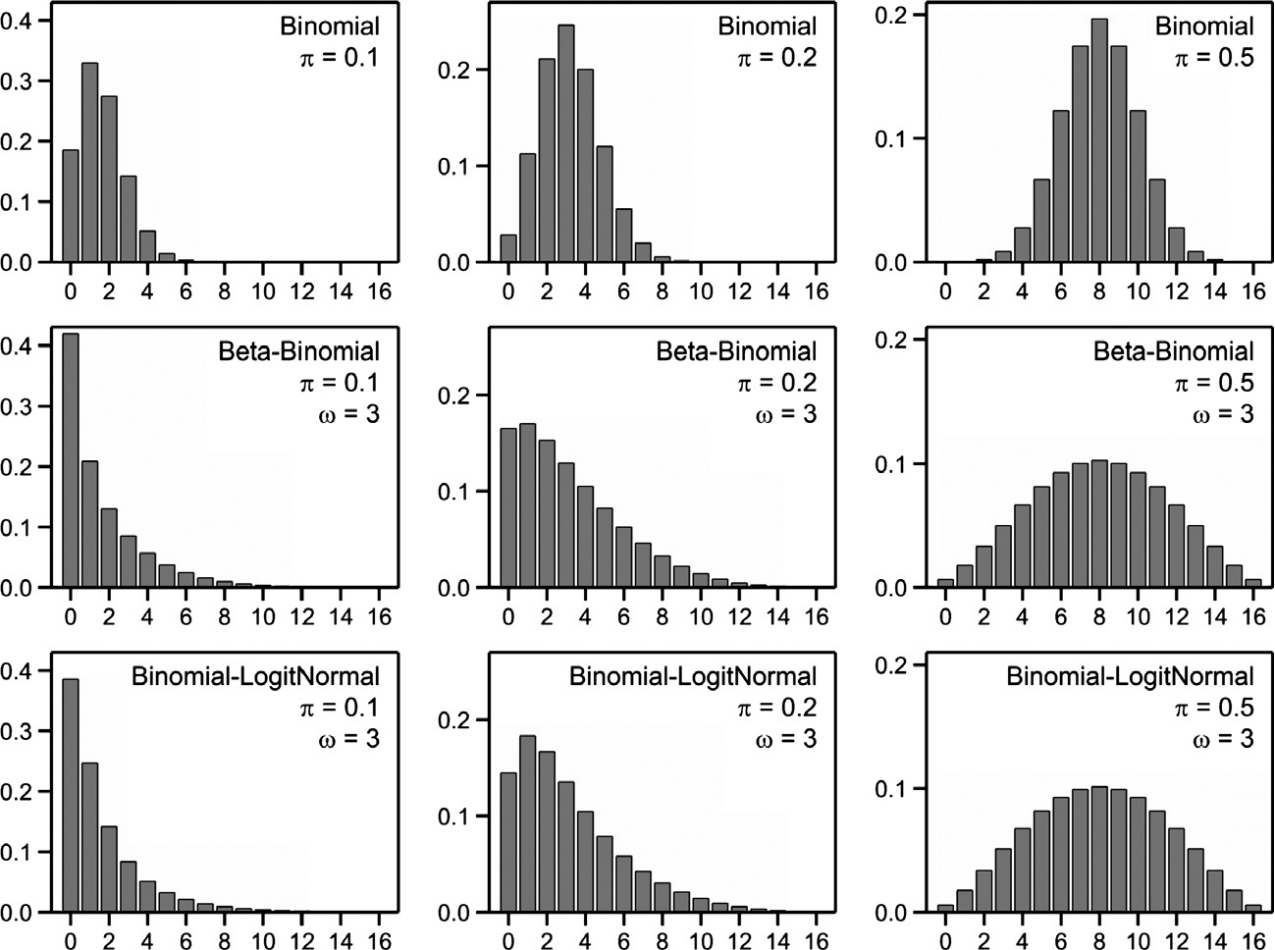
Examples of probabilities of statistical distributions for presence/absence data for *n* = 16 with mean n*π* and variance *ωnπ* (1 −*π*). For the binomial distribution, *ω* = 1.

In field experiments the presence or absence of an organism might be recorded rather than counting the organism. The response *X* might then be the number of plants on which a specific organism is present for each experimental unit. Assuming independence between the *n* plants and a fixed presence probability *π* the response follows a binomial distribution. The mean of the binomial distribution is given by *nπ* and the variance equals *nπ*(1 − *π*). Examples of the binomial distribution are given in Figure 2.

Overdispersion arises by assuming that the presence probability itself, now called *Z*, follows some statistical distribution with mean *π* and some variance *τ*^2^. It follows that the marginal mean of *X* itself equals *nπ* and the variance equals *nπ*(1 −*π*) +* n*(*n* − 1)*τ*^2^ which is larger than the variance of the binomial distribution. A popular choice for *Z* is the beta distribution which results in the so-called beta-binomial distribution. An alternative is to assume that the logit transform of *Z* follows a normal distribution. Details of both distributions are given below.

The beta-binomial distribution arises when *Z* follows a Beta distribution which is defined on the interval (0,1). A convenient re-parameterization results in a mean *nπ* and variance *nπ*(1 − *π*)[1 + (*n* − 1)*φ*]. When the number of binomial trials is equal across experimental units, the term between squared brackets is constant and the variance of the beta-binomial distribution is then proportional to the binomial variance. In this case data can be easily analyzed by the quasi likelihood approach, similar to the analyses for the overdispersed Poisson distribution. Some examples of the beta-binomial distribution are given in Figure 2. This shows that the range of possible outcomes is extended as compared to the binomial distribution. However for very large values of *ω* the distribution becomes bath-tub like with large probabilities for outcomes 0 and *n* and small probabilities for intermediate values.

An alternative is to assume that *Z* follows a logit-normal distribution. This is equivalent to the introduction of a normally distributed random effect on the scale of the linear predictor in logistic regression. For obvious reasons this distribution can be termed binomial-logitnormal. Unfortunately the mean and variance of the logit-normal distribution cannot be written in analytical form, and this is thus also the case for the binomial-logitnormal distribution itself. Probabilities can be obtained by integrating out the random effect by Gauss–Hermite quadrature. Some examples of this distribution are given in Figure 2; the parameters which are used in this figure are such that the mean and variance of the distribution are given by *nπ* and *ωnπ*(1 − *π*). This shows that for *π* = 0.5 there is hardly a difference with the beta-binomial distribution, while for smaller values of *π* the binomial-logitnormal distribution has somewhat smaller zero probabilities.

## Excess-Zeros Distributions

Although the overdispersed count distributions have a larger zero probability than the corresponding Poisson or binomial distribution, in practice the number of zero observations can still be larger than predicted by the count distribution. This is termed excess-zeros or zero-inflation. Examples of situations with excess-zeros are given by Cunningham and Lindenmayer (2005), Sileshi (2008) and Lewin et al. (2010). Failure to account for zero-inflation in a statistical analysis may results in biased estimation of environmental effects of GM plants. A common model for zero-inflation assumes that a proportion *δ* of the experimental units have a structural zero and the remaining proportion (1 − *δ*) of units follows one of the count distributions given above. The zero-inflated distribution for the resulting count *Y* is then given by

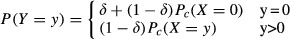
in which *P*_*c*_(*X* = *x*) is the distribution of the counts. Note that the probability of observing a zero is given by the probability *δ* of obtaining a structural zero plus the probability of obtaining a zero by chance. Having a lot of zeros in itself does not necessarily mean that a zero-inflated model is needed. An examples of this is given by the negative binomial distribution in Figure 1.

The mean of a zero-inflated Poisson distribution equals *μ*(1 − *δ*) and its variance equals *μ*(1 − *δ*)(1 + *δμ*). Regression models based on the zero-inflated Poisson distributions were introduced by Lambert (1992) who considered simultaneous modeling of *μ* and *δ* which are related to possibly different sets of covariates. Greene (1994) brought regression modeling to the zero-inflated negative binomial distribution. Hall (2000) and Vieira et al. (2000) seem to be the first papers which employ a zero-inflated binomial model. Finally, Cheung (2006) uses a zero-inflated beta-binomial model to analyze cognitive function test scores of Indonesian children.

## Statistical Simulation Model

Having defined possible probability distributions for counts and presence/absence data, the other elements of field testing of environmental effects of GM plants on NTOs can be introduced. These elements are summarized in Table 2.

**Table 2:** Elements of the statistical simulation model.

Element	Possible choices
Distribution of counts	Poisson, overdispersed Poisson, negative binomial, Poisson-lognormal, binomial, betabinomial, binomial-logitnormal
Excess-zero counts	No/yes
Design	Completely randomized, randomized blocks, number of replications
Additional varieties	Number of additional varieties or treatments in addition to the GM plant and its comparator
Reference varieties	Number of reference varieties which represent a population
Trial	Single trial, multiple trials, site × year trials
Measurement	Single time point, repeated measures (constant, linear or quadratic in time, autocorrelation)
Parameters	Parameter values for all the count distributions, for example, a mean and an excess-zero probability for each variety

### Block effects and the transformed scale

An experiment will minimally consist of a GM plant and its comparator. When these are compared in a single trial without blocking, with only a single measurement per experimental unit and no excess-zeros, simulation of such an experiment only requires specification of the mean *μ* of the count distribution for both the GM plant and the comparator. In addition, when overdispersion is required, a common dispersion parameter must be specified. Adding blocking to this experiment requires a random blocking effect. It is natural and common to introduce blocking effects for counts on the log scale, that is,

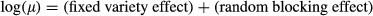


as this ensures that the mean *μ* of the count is always positive. Note that this also requires that the variety effect of the GM plant and the comparator is specified on the log scale. Likewise, the logit transformation can be used to specify the probability of success *π* for the presence/absence data and/or the excess-zeros probability *δ*, for example,

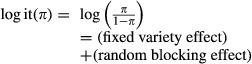


This ensures that probabilities are always in the interval (0,1). So in the simulation model, all effects are introduced on the natural log scale for counts and on the logit scale for probabilities.

When an experiment with blocking is required, it is assumed that block effects follow a normal distribution with some variance. For each new block, a block effect is simulated according to this distribution and added to the variety effect on the transformed scale. Note that when an excess-zero distribution is used, a block variance must be specified for both the count or presence/absence distribution as well as for the excess-zero probability.

### Additional varieties and reference varieties

In addition to the GM plant and the comparator, other varieties can be introduced in the experiment. These might be other comparators or other GM plants, or alternatively, the GM plant and/or comparator itself which receive different agronomical treatments with for instance herbicides. Although the latter are not varieties but rather treatments, for simplicity, these are also termed additional varieties in the simulation model. For each of the additional varieties, a variety effect on the transformed scale must be specified.

A special case is an experiment in which the GM plant and its comparator are to be compared with a group of reference varieties which are assumed to have a history of safe use (Van der Voet et al., 2011). In this case, the individual reference varieties themselves are not of interest, but they are rather used to derive baselines or equivalence limits. The reference varieties in the experiment might thus be considered as representing a population of reference varieties. It is then natural to assume that the variety effect of each reference variety is drawn from a statistical distribution. For convenience, a normal distribution is used for this. The difference between additional and reference varieties is that for each additional variety, a fixed variety effect must be specified, whereas for reference varieties, only a common variety effect and an associated variance must be specified.

At this stage, it is instructive to go through the different steps in the statistical simulation model for a hypothetical small experiment. Suppose a GM plant, its comparator and three reference varieties are to be compared in a randomized block experiment with 2 blocks. A zero-inflation Poisson distribution is used with the following parameters for the varieties:

**Table:** 

Variety	Effect count	Variance count	Effect zero	Variance zero
GM plant	0.4	–	−0.3	–
Comparator	0.5	–	−0.2	–
Reference varieties	1	1	−0.8	0.5

First the count effect on the log scale of the three reference varieties is drawn from a normal (1,1) distribution; suppose that this results in random draws 0.8, 0.9 and 1.2. Secondly, the excess-zero probability effect on the logit scale for the reference varieties is drawn from a normal (–0.8, 0.5) distribution; suppose that this results in –1.2, –0.9 and –0.6. Furthermore, assume that the between block variance for the counts equals 0.1 with random draws −0.4 and 0.1 for the two blocks and that the between block variance for the excess-zero probabilities equals 0.01 with random draws −0.1 and 0.2. The mean count *μ* and excess-zero probability *δ* for every plot in the experiment are then given by

**Table:** 

Variety	*μ* Block 1	*μ* Block 2	*δ* Block 1	*δ* Block 2
GM plant	exp(**0.4**–0.4)	exp(**0.4 **+** **0.1)	logit^−1^**(**−**0.3**–0.1)	logit^−1^**(**−**0.3 **+** **0.2)
Comparator	exp(**0.5**–0.4)	exp(**0.5 **+** **0.1)	logit^−1^**(**−**0.2**–0.1)	logit^−1^**(**−**0.2 **+** **0.2)
Reference 1	exp(0.8–0.4)	exp(0.8 + 0.1)	logit^−1^(−1.2–0.1)	logit^−1^(−1.2 + 0.2)
Reference 2	exp(0.9–0.4)	exp(0.9 + 0.1)	logit^−1^(−0.9–0.1)	logit^−1^(−0.9 + 0.2)
Reference 3	exp(1.2–0.4)	exp(1.2 + 0.1)	logit^−1^(−0.6–0.1)	logit^−1^(−0.6 + 0.2)

These values are then used to generate a count for each plot. In a simulation study, typically many datasets are simulated with the same settings. For each dataset, new reference variety effects and new blocking effects are simulated, so only the bold values remain the same for each simulated dataset.

### Repeated measurements

Non-target organisms at the same experimental unit are frequently sampled at different points in time, see for example Head et al. (2005). The simulation model assumes that time points are equidistant, that is, 1, 2, …, *T*. There are many possible patterns in time. The simulation model accommodates constant, linear or quadratic time patterns, all on the transformed scale, and this can be set separately for the mean of the counts and for the excess-zero probability. Moreover, the repeated observations on the same experimental unit can be independent or can be correlated. The mean *μ*_t_ of each variety at time point *t*, assuming no block effects, is given by log(*μ*_*t*_) = *f*_*p*_(*t*) + *v*_*t*_, where *f*_*p*_(*t*) is a polynomial up to order *p* = 2. The extra random effect *v*_*t*_ specifies the correlation between repeated measures. Absence of a time effect is simply given by *f*_0_(*t*) = *β*_0_, a linear time effect by *f*_1_(*t*) = *β*_0_ + *β*_1_*t* and a quadratic time effect by *f*_2_(*t*) = *β*_0_+*β*_1_*t*+*β*_2_*t*^2^. An alternative parameterization for the second-order polynomial with more meaningful parameters is given by *f*_2_(*t*) = *β*_max_ − (*t* −*β*_opt_)^2^/(2*β*_tol_), where the minimum or maximum *β*_max_ is attained for the optimal time point *β*_opt_ and the parameter *β*_tol_ represents the width of the quadratic curve, also called the tolerance. This latter parameterization is used in the simulation tool. For a positive tolerance, the parabola has a maximum, while for a negative tolerance, it has a minimum. The vector of random effects ***v*** = (*v*_1_, …, *v*_*T*_) is assumed to follow a multivariate normal distribution, that is, 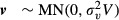 where *V* is a *T* x*T* symmetric correlation matrix. The simulation tool implements three options for the random effects:

1. No extra variability as given by 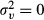.
2. Equal correlation across time by setting *V*_*kk*_ = 1 and *V*_*kl*_ = *ρ* for *k* ≠ *l*
3. Autoregressive correlation across time by setting *V*_*kl*_ = *ρ*^|*k*−*l*|^

Note that the second and third option involves an overdispersion mechanism. Consequently, an equal correlation across time with *ρ* = 0 combined with the Poisson distribution is equivalent with the Poisson-lognormal distribution with no extra variability across time. A combination with, for example, the negative binomial distribution however involves two levels of overdispersion. The simulation tool requires specification of the *β* parameters for the GM plant and its comparator and also for the additional varieties. When excess-zeros are desired, a different set of *β* parameters must be specified for the excess-zero probability model. For the reference varieties, each *β* parameter is drawn from a normal distribution with specified mean and variance.

### Multiple trials

Multiple field trials across environments fall into two main classes. One in which there is no further structure across trials, for example, when trials are conducted at different sites, and secondly, when the trials follow a site × year structure. In the first case, there might be random trial effects such that trials will vary in their level of response without affecting differences between varieties. This is in addition to random block effects within trials. In the second case, experiments, carried out at different sites, are replicated for a limited number of years. Then, random site and random year effects are conceivable as well as a random site by year interaction effect. Note that these random effects are added to the other effects on the transformed (log or logit) scale.

In addition to additive multiple trial effects on the transformed scale, variety effects might be different from trial to trial, from site by site or from year to year. This can be termed genotype by environment interaction. This interaction can be accommodated by additional random effects which operate on variety effects. Instead of a fixed variety effect, for e.g. the GM plant, in each separate trial, the variety effect is drawn from a normal distribution with some mean and variance. For the reference varieties, there are two stages of simulation. In the first stage, a reference mean, say M, for the trial is drawn from a normal distribution with specified mean and variance. In the second stage, the effects of the reference varieties in that specific trial are simulated from a normal distribution with mean M and some other specified variance. Similarly for site × year trials, three variance components can be distinguished for every variety effect. In case of repeated measurements with say a quadratic time effect, all the time effect *β* parameters have their associated variance components and many parameters need to be specified. Moreover, the same statistical model can be specified for the excess-zero probability.

## Examples of Simple Simulations

The simulation model was implemented in a software tool, available as Supplementary Material, and was used to perform a series of simulations to demonstrate various aspects which can be studied by means of the tool. To this end, single, completely randomized trials were simulated to assess properties of statistical difference and equivalence testing. In addition to the GM plant and its comparator, one additional variety was included in every simulation and the simulated response was a count. The mean of the comparator and the additional variety was assumed to be equal to say *μ*, and the mean of the GMO is denoted by *θμ* such that *θ* is the multiplicative difference between the GM plant and the comparator. The following levels of replication *N* were employed: *N* = 4, 6, 8, 10, 15, 20, 30 and 40. A two-sided test with a significance level of *α* = 0.05 was used throughout. In each example, 1000 datasets were simulated for every parameter combination.

### Power of difference test for the negative binomial distribution

The power of a likelihood ratio test for the difference between a GM plant and its comparator was studied. Data were simulated according to the negative binomial distribution. All combinations of the following values were used:

1. *μ*   1, 2, 5, 10, 20, 40
2. *θ*   1, 1.2, 1.4, 1.6, 1.8, 2.0
3. *ω*   0.25, 0.5, 1

**Figure 3:**
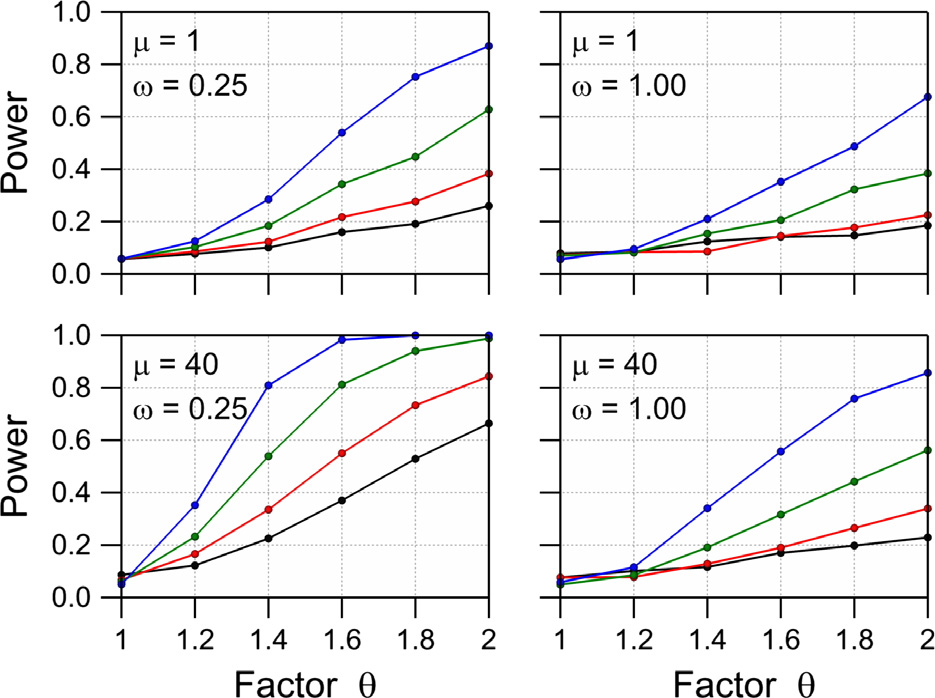
Power of a likelihood ratio difference test with *α* = 0.05 for the negative binomial distribution with dispersion parameter *ω*, a mean *μ* for the comparator and a mean *θμ* for the GM plant for replication levels *N* = 6 (black), 10 (red), 20 (green), and 40 (blue).

The negative binomial distribution was fitted to each dataset, first under the restriction that the mean of the GMO equals the mean of the comparator and secondly, without this restriction. A likelihood ratio test statistic is then given by twice the difference between the log likelihoods of the two models. The large sample distribution of this test statistic is 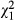, and this distribution was used to calculate *P* values. The (simulated) power of the difference test is then given by the fraction of the 1000 simulated datasets for which the null hypothesis of no difference is rejected. Examples of resulting power curves are given in Figure 3. The number of replications required to detect a multiplicative difference *θ *= 2 between the mean of the GM plant and the comparator with probability 0.80 is given in Table 3 for the various values of *μ* and *ω*. These values were interpolated from the values of *N* which are used in the simulation. As expected, the power is larger when there is less overdispersion (smaller values of *ω*) and when the mean of the distribution is large.

**Table 3:** Number of replications needed to obtain a significant difference test with probability 80% when the quotient of the mean of the GMO and the comparator equals *Θ* = 2 for data which have a negative binomial distribution with mean *μ* for the comparator, mean *Θμ* for the GM plant, and dispersion parameter *ω*.

*ω*	*μ* = 1	*μ* = 2	*μ* = 5	*μ* = 10	*μ* = 20	*μ* = 40
0.25	29	21	13	10	9	9
0.50	≥40	27	21	19	17	16
1.00	≥40	≥40	37	35	33	32

### Sensitivity analysis for the negative binomial distribution

**Figure 4:**
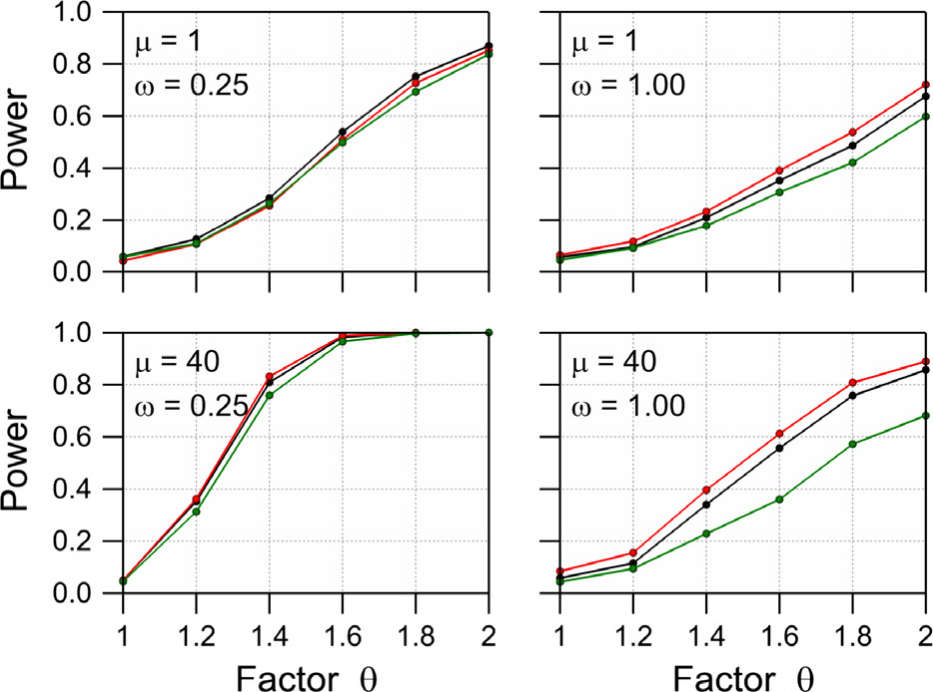
Power of a likelihood ratio difference test with *α* = 0.05 for negative binomial data with dispersion parameter *ω*, a mean *μ* for the comparator, and a mean θ*μ* for the GM plant for replication level *N* = 40 when analyzed employing a negative binomial model (black), a quasi-Poisson model (red), and a log transformation (green).

The true underlying distribution of field count data is generally not known. Furthermore, especially for small samples, it is difficult to discriminate between the various statistical distributions. To assess the sensitivity to the assumed distributional form, each simulated negative binomial dataset was also analyzed by two alternative models. The first alternative employs the quasi-Poisson approach in which the likelihood ratio test was scaled by the mean deviance under the full model, whenever this mean deviance is larger than 1. The second alternative first log transforms the count data, after the addition of 0.5, whenever there is at least one zero in the data, followed by an analysis of variance. Examples of power curves for replication *N* = 40 for the three models are given in Figure 4. This seems to suggest that the power of the negative binomial and the quasi-Poisson analysis are very similar and that the power of the log-transform approach is somewhat smaller especially for the combination of a large mean *μ* and a large dispersion parameter *ω*.

### Properties of equivalence test for the Poisson distribution

**Figure 5:**
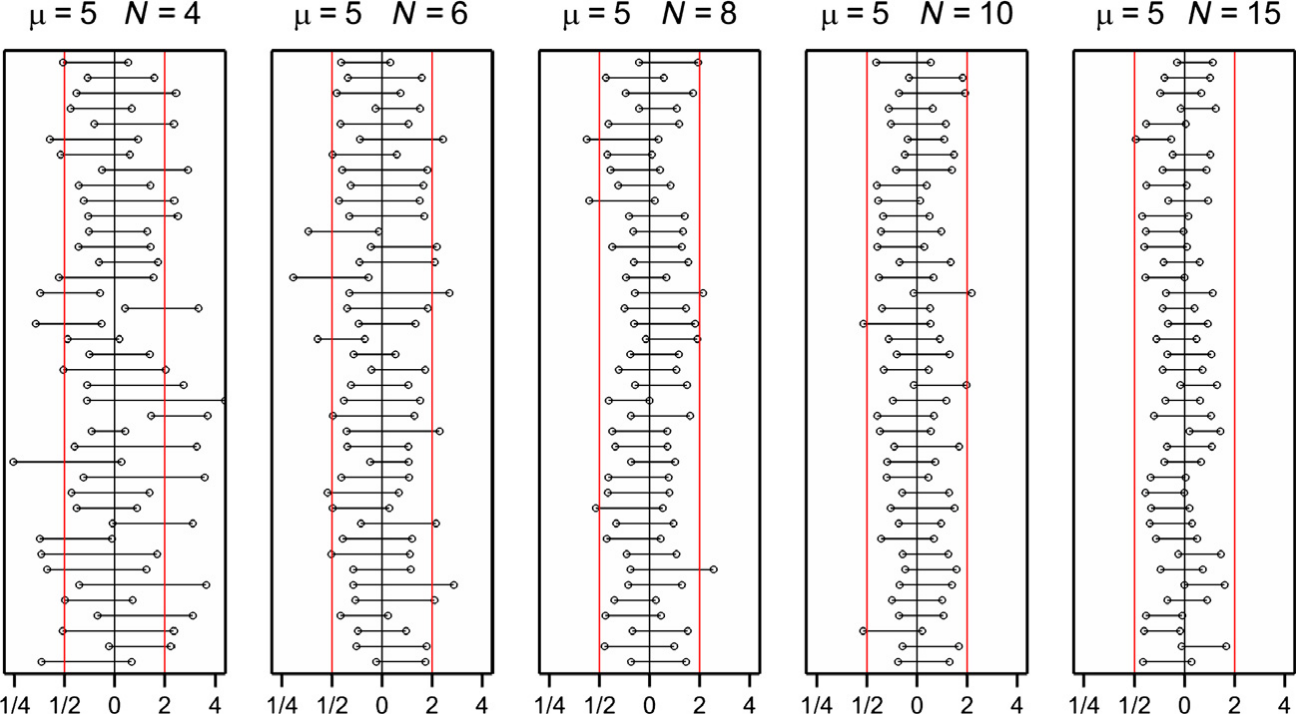
95% likelihood ratio confidence intervals for the ratio of the Poisson means of the GM plant and the comparator when the underlying mean of both is *μ* = 5 and various numbers of replication *N*. The red vertical lines denote the artificial equivalence limits set at 1/2 and 2.

Properties of the TOST approach to equivalence testing were assessed for count data which were simulated according to the Poisson distribution. The null hypothesis of non-equivalence is rejected in favor of equivalence when the confidence interval completely lies in the interval determined by fixed lower and upper equivalence limits. The same simulation setting as in the first simulation was used However, as the Poisson distribution was employed to simulate data there is no over dispersion. Hypothetical equivalence limits of ½ and 2 were employed to perform equivalence testing. A 95% likelihood ratio confidence interval for the ratio of the GMO mean and the comparator mean was calculated for each simulated dataset. The number of times this interval lies within the equivalence interval (½, 2) can then be counted. As an example, the confidence interval for 40 simulated datasets is given in Figure 5 with *μ* = 5 for both the GMO and the comparator, so *θ* = 1, and for various values of the number of replications *N*. In this case, the GMO and comparator have equal means and are thus theoretically equivalent. However, for small numbers of replications, the confidence intervals frequently cross the equivalence limits implying that the null hypothesis of non-equivalence is not always rejected.

The number of replications required to detects a quotient of *δ* between the GM plant and its comparator, as well as the number of replications required to reject the null hypothesis of non-equivalence with equivalence limits of ½ and 2 is given in Table 4. This clearly shows the asymmetry in the requirements for a difference test and an equivalence test. As the multiplicative difference *θ* increases, the number of replicates for a difference test decreases, while those for an equivalence test increases.

**Table 4:** Number of replications needed to obtain a significant difference test or to reject the hypothesis of non-equivalence with limits ½ and 2, with probability 80% when the quotient of the mean of the GMO and the comparator equals *Θ* for data which have a Poisson distribution with mean μ for the comparator and mean *Θ*μ for the GM plant.

*Θ*	*μ* = 1	*μ* = 2	*μ* = 5	*μ* = 10	*μ* = 20	*μ* = 40
Replications for Difference test, *Θ*						
1.0	–	–	–	–	–	–
1.2	≥40	≥40	≥40	≥40	22	12
1.4	≥40	≥40	24	13	6	≤4
1.6	≥40	28	12	6	≤4	≤4
1.8	36	18	7	≤4	≤4	≤4
2.0	24	12	5	≤4	≤4	≤4
Replications for Equivalence test, *Θ*						
1.0	≥40	20	8	5	≤4	≤4
1.2	≥40	26	11	6	≤4	≤4
1.4	≥40	≥40	20	10	5	≤4
1.6	≥40	≥40	≥40	23	12	6
1.8	≥40	≥40	≥40	≥40	≥40	23
2.0	–	–	–	–	–	–

### The effect of excess-zeros

**Figure 6:**
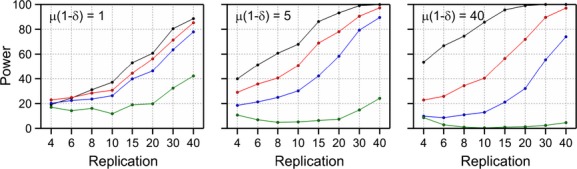
Power of a likelihood ratio difference test with *α* = 0.05 for negative binomial data with overdispersion parameter *ω* = 0.25 and additional excess-zeros with probability *δ* = 0 (black), 0.1 (red), 0.2 (blue), and 0.5 (green). The comparator has mean *μ*(1 −*δ*), and the GM plant has a mean of 2*μ*(1 − *δ*).

To evaluate the effect of excess-zeros on the power of the ordinary likelihood ratio test, a separate simulation with the excess-zero negative binomial distribution was executed. Again, a single trial without blocking with a single measurement was assumed. Furthermore, a multiplicative difference of *θ* = 2 was used between the GM plant and the comparator. The excess-zero probability was set to *δ* = 0, 0.1, 0.2 and 0.5. The mean (1−*δ*)*μ* of the excess-zero distribution was set to 1, 5 and 40 ensuring that the means of the distributions are identical for different values of *δ*. The data were analyzed with the negative binomial distribution as if there were no excess-zeros. The power for different levels of replication is given in Figure 6. This indicates that for small means and small excess-zero probabilities, the power is not much affected. However, for larger means, there can be a considerable decline of the power. For an excess probability of *δ* = 0.5 and larger means, the resulting distribution has a spike at zero in combination with larger values with not very much in between. In such a situation, the estimate of the dispersion parameter becomes very large so as to “catch” both the zeros and the larger observations. Consequently, the distinction between the means of the comparator and the GMO disappears resulting in very low power values. In such a case, the data should be analyzed by means of an excess-zero distribution.

### Repeated measurements

**Figure 7:**
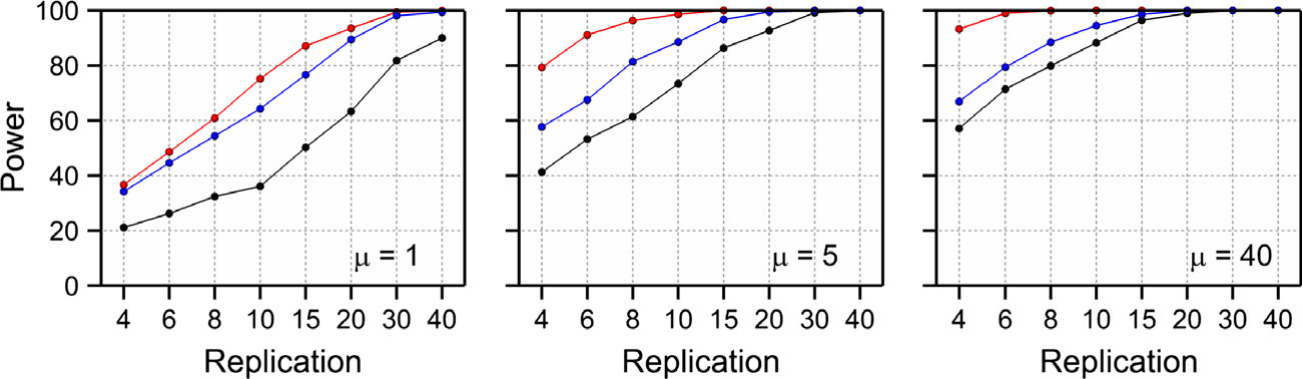
Power of a likelihood ratio difference test for Poisson-lognormal data with overdispersion parameter *ω* = 0.25 and a single observation (black), the sum of 5 independent observations (red), and the sum of 5 dependent observations (blue). The mean of both the comparator and the GM plant follows a quadratic polynomial on the log scale with a maximum mean count of *μ* for the comparator and 2*μ* for the GM plant (see text).

In field studies, observations on the same experimental units are frequently carried out on different points in time. The question then arises whether it is more fruitful to increase the number of observations in time or to increase the number of experimental units to achieve a certain power. Consider the situation where the mean count of a species follows a second-order polynomial on the log scale with a maximum *μ* at *t* = 0 and a value of 0.6*μ* at *t* = ± 7 days. This defines a second-order polynomial on the log scale with parameters *β*_opt_ = 0, *β*_tol_ = 47.96 and *β*_max_ = log(*μ*). According to the polynomial, the mean count at *t* = ± 14 days equals 0.13*μ*. Data were simulated with the Poisson-lognormal model with a dispersion parameter *ω* = 0.25. This was carried out for the situation in which the comparator has a maximum mean count of *μ* and the GM plant a maximum of 2*μ*, so *θ* = 2. Three situations are simulated: a) a single observation for each experimental unit at *t* = 0, b) repeated observations at *t* = −14, −7, 0, 7 and 14 assuming that these observations are independent and c) repeated observations at the same five points in time but now assuming an autoregressive correlation across time for the five random effects on the log scale with correlation *ρ* = 0.8. Independent observations in time are included as a limiting case because in practice, it is unlikely that repeated observations on the same experimental unit are independent. In cases b) and c), observations on the five time points are summed for every experimental unit, and in all cases, the negative binomial distribution, which has the same variance function as the Poisson-lognormal, was used for analyzing the data. The power curve of the difference test for various replication levels is given in Figure 7. For *μ* = 1, there is a large benefit of repeated measurements for both the independent and the correlated counts in time. This benefit becomes smaller for larger *μ* for the correlated counts, possibly to a point that it is more efficient to increase the replication level rather than sampling at different points in time. This will however also depend on the size of the correlation. Only a minority of the papers listed in Table1 explicitly considered this issue in analyzing data from field experiments.

The confidence interval for the difference parameter on the log scale was used to perform equivalence testing. The number of replications required to reject the null hypothesis of non-equivalence with equivalence limits of 1/3 and 3 is given in Table 5. This also shows that, for the correlation case, the advantage of multiple sampling times becomes smaller as the mean increases.

**Table 5:** Number of replications needed to reject the hypothesis of non-equivalence with limits 1/3 and 3, with probability 80% using a two-sided test for the repeated measurements simulation, see text.

Maximum mean *μ*	Single observations	Multiple dependent	Multiple independent
1	≥40	≥40	39
2	≥40	33	23
5	39	25	14
10	30	20	10
20	26	20	9
40	24	19	8

## Discussion

This paper describes a general framework for simulating data typically encountered in environmental risk assessment of genetically modified plants. It focuses on field testing of GM plants to assess and compare their potential adverse effects on non-target organisms. Based on such field trials, the assessment includes the use of statistical difference and equivalence testing. It is important to know the statistical properties of such tests, for example, the power and robustness of a test and whether the test has the correct significance level. The EFSA guideline (EFSA, 2010) for environmental risk assessment of GM plants for instance requires that each field trial should have sufficient replication to be able to yield a stand-alone analysis if required. Such and other statistical issues related to the assessment can best be researched by means of a statistical simulation model.

Limits of concern are required for equivalence testing, but these may be difficult to specify for NTO experiments. They can of course be set to a fixed value by authorities, such as a 20% increase or decrease (Hothorn and Oberdoerfer, 2006), but this remains largely arbitrary. Alternatively, a set of reference varieties can be included in the same comparative experiment to allow comparison with the GM plant grown under the same conditions (van der Voet et al., 2011). The natural variation, in, for example, counts of NTOs, between the reference varieties can then be used to set limits of concern.

The simulation model described in this paper can be used to generate data for various endpoints having different statistical distributions. Typical environmental data of non-target organisms are counts or presence/absence data. The basic distribution for counts is the Poisson distribution, while presence/absence data are commonly modeled by the binomial distribution. Taylor's power law (Taylor, 1961) suggests that overdispersion, relative to the Poisson distribution, is likely to be normal in environmental studies. Statistical distributions for this phenomena are the overdispersed Poisson, negative binomial and Poisson-lognormal distribution for counts; these have much wider tails and also a larger zero probability. Likewise, overdispersed distributions for binomial data are the beta-binomial and the binomial-logitnormal distribution, which seem to be quite similar for a wide range of parameter values. In addition to overdispersion, excess-zeros might frequently be encountered especially for rather rare species (Cunningham & Lindenmayer, 2005). Failure to account for this in the analysis may result in biased estimation of ecological effects (Sileshi, 2008). The simulation model therefore also addresses zero-inflation for every distribution just described.

Field trials for environmental risk assessment may be too small to discriminate between the various statistical distributions. For example, a large number of zeros can be due to structural zeros and thus zero-inflation or can be due to heavy clumping of individuals which gives rise to a negative binomial distribution with a large dispersion parameter. The simulation model can be used to assess the robustness of statistical models to digressions from the model. For instance, an analysis according to Taylor's power law, or possible an analysis of log counts using the normal distribution, might be a good comprise for the various overdispersed count distributions. This can also be researched by means of the simulation model.

In many experiments, non-target organisms are sampled at different points in time. The simulation model accommodates this by the option to specify a constant, linear or quadratic pattern in time on the log- or logit-transformed scale. Moreover, repeated measures at the same plot might exhibit autocorrelation which can be modeled by a random effect with equal correlation across time or by an autoregressive process. This gives the opportunity to study various endpoints such as the summed counts over a specified period of time, the time of first occurrence of a species, the time at which maximal abundance of a species occurs, or even the repeated measures themselves.

Genotype by environment interaction is an important issue in the admission of GM plants. In the European context, the EFSA guideline (EFSA, 2010) also focuses on the receiving environment of the GM plant, assuming that different bio-geographical zones, in terms of meteorological, ecological and agricultural conditions, may comprise different risks of growing GM plants. To develop appropriate statistical methods to handle genotype by environment interaction in studies over multiple bio-geographic regions and under varying agronomical conditions, a simulation tool is indispensable. The interaction is accommodated by the simulation model by additional random effects which operate on variety effects. Instead of a fixed variety effect, for e.g. the GM plant, in each separate trial (or environment), the variety effect, on the transformed scale, is drawn from a normal distribution with some mean and variance. This ensures that the difference between the GM plant and its comparator does have a common basis across trials, but might be varying from trial to trial. As an alternative, the simulation model also caters for a site by year interaction in which the “environments” are now structured by sites and years.

All these elements are implemented into a single computer program which can handle non-normal distributions for counts and absence/presence data possibly with excess-zeros, accommodates reference varieties in addition to the GM plant and its comparator, employs completely randomized or randomized block experiments, enables genotype by environment interaction by adding random variety effects, and finally repeated measures in time following a constant, linear, or quadratic pattern in time possibly with some form of autocorrelation. The computer program is available as Supplementary Material to this paper.

Although the tool is quite comprehensive, there are certain restrictions. It is not possible to define different dispersion parameters for different varieties or for different trials. This might be useful when one would like to keep the coefficient of variation constant across varieties rather than the dispersion parameter. Simulation by means of Taylor's power law is not implemented as there is no distribution associated with this law. In the current simulation model, it is not possible to have a linear effect in time for one variety and a quadratic time effect for another variety, but if needed, pseudo-linear behavior can be obtained by specific choices of the quadratic parameters. Also the time dependence is assumed to be equal across varieties. Split-plot experiments, with for example agronomical treatments on the main plot level and varieties on the subplot level, are not supported.

The tool was used to perform a series of simple preliminary simulations to demonstrate various aspects which can be studied by means of the tool. It is shown that the tool can be used for a prospective power analysis for both difference testing and equivalence testing. A simple sensitivity analysis seems to imply that, for simple experiments, the assumed distributional form may not be very critical. The effect of a small proportion of excess-zeros might be small when the mean count itself is also small. Finally, it appears that the benefit of repeated measures, assuming autoregressive correlation in time, becomes smaller when the mean increases.

Based on the simulation model as described in this paper, further work is needed to develop a protocol for prospective power analysis when designing field experiments for environmental non-target effects of GM plants. Requiring the use of such a protocol could avoid literature reports where the conclusion of non-significant differences between GM and non-GM plants is not accompanied by a report on the statistical power of the field experiment.
